# Supplementary material for: Agronomic, Physicochemical, Aromatic and Sensory Characterization of Four Sweet Cherry Accessions of the Campania Region
Source: Plants (Basel). 2023 Jan 30;12(3):610. doi: 10.3390/plants12030610 (PMC9921131; doi:10.3390/plants12030610)
Supplement: Supplementary file 1 [file plants-12-00610-s001.zip › plants-2157883-supplementary.pdf]

Table S1. The International Union for the Protection of New Varieties of Plants (UPOV) of the four sweet cherry accessions (Mulegnana Riccia, Mulegnana Nera, Montenero and Limoncella) compared to two standard cultivars (Lapins and 'Ferrovia).

UPOV descriptors	Mulegnana Riccia	Mulegnana Nera	Montenero	Limoncella	Lapins	Ferrovia
Tree: vigor	Medium	Medium	Medium	Medium	Medium	Medium
Tree: habit	Spreading	Spreading	Spreading	Semi-upright	Drooping	Semi-upright
Tree: branching	Strong	Medium	Medium	Strong	Medium	Medium
Young shoot: anthocyanin coloration of apex	Medium	Medium	Medium	Weak	Medium	Medium
Young shoot: pubescence of apex	Medium	Medium	Medium	Medium	Medium	Medium
One-year-old shoot: length of internode	Normal	Normal	Normal	Normal	Normal	Normal
One-year-old shoot: number of lenticels	Medium	Medium	Medium	Medium	Medium	Medium
One-year-old shoot: thickness	Medium	Medium	Medium	Medium	Thick	Medium
Leaf blade: length	Medium	Long	Long	Long	Long	Medium
Leaf blade: width	Medium	Medium	Broad	Medium	Medium	Medium
Leaf blade: ratio length/width	Medium	Medium	Medium	Medium	Large	Medium
Leaf blade: intensity of green color of upper side	Medium	Medium	Medium	Medium	Medium	Medium
Leaf: length of petiole	Long	Long	Long	Long	Long	Long
Leaf: ratio length of blade / length of petiole	Medium	Large	Medium	Medium	Small	Medium
Leaf: presence of nectaries	Present	Present	Present	Present	Present	Present
Nectaries: color	Light red	Dark red	Light red	Light red	Light red	Light red
Flower: diameter	Medium	Large	Medium	Small	Medium	Medium
Flower: shape of petal	Broad obovate	Circular	Medium obovate	Medium obovate	Broad obovate	Broad obovate
Flower: arrangement of petals	Overlapping	Free	Intermediate	Free	Overlapping	Intermediate
Fruit: Size	Medium	Medium	Large	Medium	Large	Large
Fruit: Shape	Cordate	Reniform	Reniform	Cordate	Cordate	Cordate
Fruit: Pistil end	Flat	Flat	Flat	Flat	Flat	Pointed
Fruit: Suture	Weakly conspicuous	Absent or very weakly conspicuous	Absent or very weakly conspicuous	Weakly conspicuous	Weakly conspicuous	Weakly conspicuous
Fruit: Length of stalk	Medium	Long	Medium	Long	Medium	Medium
Fruit: Thickness of stalk	Medium	Medium	Thin	Medium	Medium	Medium
Fruit: Abscission layer between stalk and fruit	Present	Present	Present	Present	Present	Present

Fruit: Color of skin	Dark red	Dark red	Red	Yellow with blush	Dark red	Dark red
Fruit: Size of lenticels on skin	Medium	Large	Small	Medium	Large	Medium
Fruit: Number of lenticels on skin	Few	Few	Many	Few	Many	Medium
Fruit: Thickness of skin	Medium	Thick	Thick	Medium	Thick	Thick
Fruit: Color of flesh	Medium red	Dark red	Medium red	Yellow	Medium red	Pink
Fruit: Color of juice	Red	Red	Red	Light yellow	Red	Red
Fruit: Firmness	Medium	Medium	Firm	Medium	Medium	Medium
Fruit: Acidity	Medium	Medium	Medium	Medium	Medium	Medium
Fruit: Sweetness	Medium	Medium	Low	Medium	Hight	Hight
Fruit: Juiciness	Strong	Medium	Medium	Strong	Medium	Medium
Stone: size	Small	Medium	Medium	Medium	Medium	Medium
Stone: shape in ventral view	Broad elliptic	Broad elliptic	Broad elliptic	Broad elliptic	Broad elliptic	Circular
Attributed Fruit: ratio weight of fruit / weight of stone	Medium	Medium	Medium	Medium	Medium	Medium
Time of beginning of flowering	Medium	Medium	Early	Medium	Medium	Late
Time of beginning of fruit ripening	Medium	Medium	Early	Early	Medium	Medium

Table S2. Volatile organic compounds (VOCs) detected of the four sweet cherry accessions (Mulegnana Riccia, Mulegnana Nera, Montenero and Limoncella) compared to two standard cultivars (Lapins and ‘Ferrovia) and their identification codes.

Metabolite	Code	^{a}Ri$_{t}$/RI$_{sp}$	^{b}ID	Metabolite	Code	^{a}Ri$_{t}$/RI$_{sp}$	^{b}ID
**Aldehydes**				**Terpenes**			
Hexanal	Ald1	1084/1086	RI/MS/S	dl-Limonene	T1	1214/1215	RI/MS/S
cis-3-Hexenal	Ald2	1148/1148	RI/MS	β-Ocimene	T2	1248/1248	RI/MS
2-Hexenal	Ald3	1242/1248	RI/MS/S	Ocymene	T3	1269/1259	RI/MS/S
Octanal	Ald4	1309/1308	RI/MS/S	Linalool	T4	1549/1549	RI/MS/S
Nonanal	Ald5	1401/1401	RI/MS/S	α-Terpineol	T5	1703/1702	RI/MS/S
Decanal	Ald6	1506/1505	RI/MS/S	Myrtenol	T6	1808/1808	RI/MS/S
Benzaldehyde	Ald7	1530/1532	RI/MS/S	Gerianiol	T7	1858/1858	RI/MS/S
Dodecanal	Ald8	1716/1713	RI/MS/S	**Esters**			
**Alcohols**				Hexyl acetate	E1	1289/1289	RI/MS/S
1-Penten-3-ol	Al1	1188/1189	RI/MS/S	2-Hexen-1-ol acetate	E2	1346/1342	RI/MS/S
1-Pentanol	Al2	1223/1222	RI/MS/S	2-Hexenyl butyrate	E3	1479/1475	RI/MS/S
3-Methyl-3-buten-1-ol	Al3	1235/1236	RI/MS/S	**Others**			
3-Methyl-2-buten-1-ol	Al4	1336/1334	RI/MS/S	6-Methyl-5-hepten-2-one	O1	1350/1348	RI/MS/S
1-Hexanol	Al5	1340/1339	RI/MS/S	3-Methylbutanoic acid	O2	1680/1680	RI/MS/S
trans 3-Hexen-1-ol	Al6	1367/1366	RI/MS/S				
cis 3-Hexen-1-ol	Al7	1374/1374	RI/MS/S				
cis 2-Hexen-1-ol	Al8	1394/1394	RI/MS/S				
1-Octen-3-ol	Al9	1446/1446	RI/MS/S				
2-Ethyl-1-hexanol	Al10	1484/1484	RI/MS/S				
1-Octanol	Al11	1498/1499	RI/MS/S				
Benzyl alcohol	Al12	1881/1882/	RI/MS/S				
Phenethyl alcohol	Al13	1871/1871	RI/MS/S				
Phenol	Al14	1882/1881	RI/MS/S				

^{a}RI$_{t}$: Relative retention indices on polar column reported in literature by www.pherobase.com; www. flavornet.org; www.ChemSpider.com; webbook.nist.gov; RI$_{sp}$: Relative retention indices calculated against n-alkanes (C_8-C_{40}) on HP-Innowax column; ^{b}Identification method as indicated by the following: RI: Kovats retention index on a on HP-Innowax column; MS: NIST and Wiley libraries spectra; S: co-injection with authentic standard compounds on the HP-Innowax column

Figure S1. Sensorial traits of the four sweet cherry accessions (Mulegnana Riccia, Mulegnana Nera, Montenero and Limoncella) compared to two standard cultivars (Lapins and 'Ferrovia).

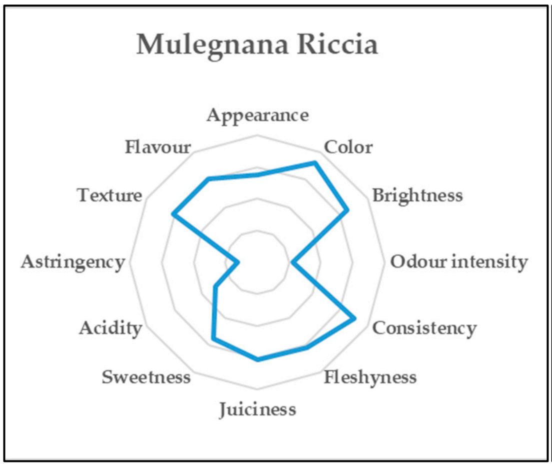

(a)

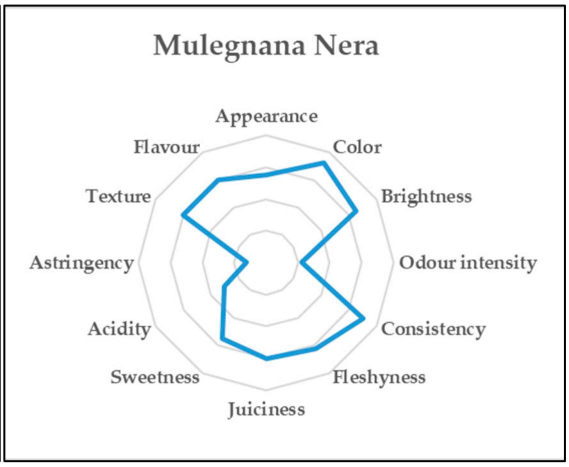

(b)

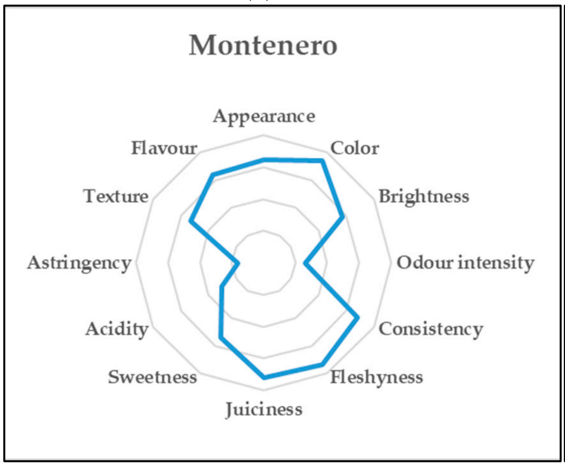

(c)

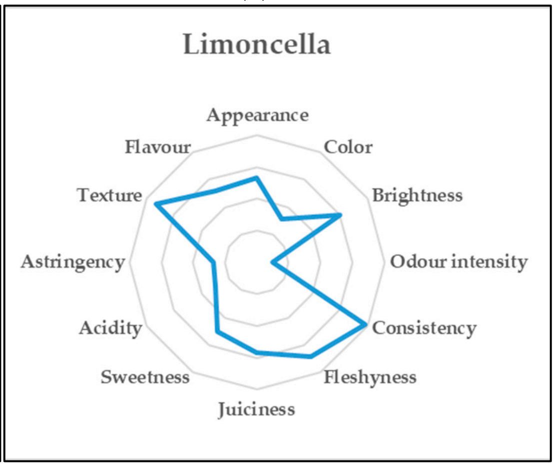

(d)

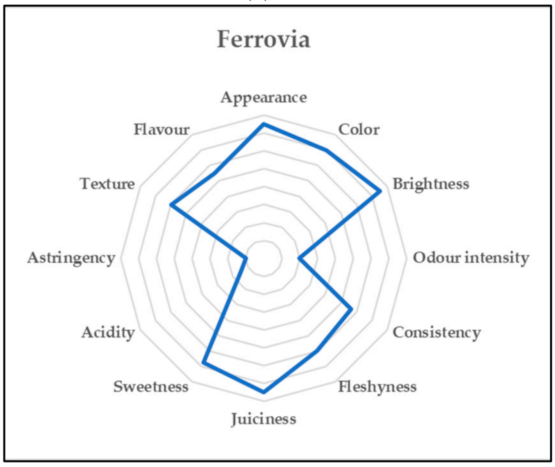

(e)

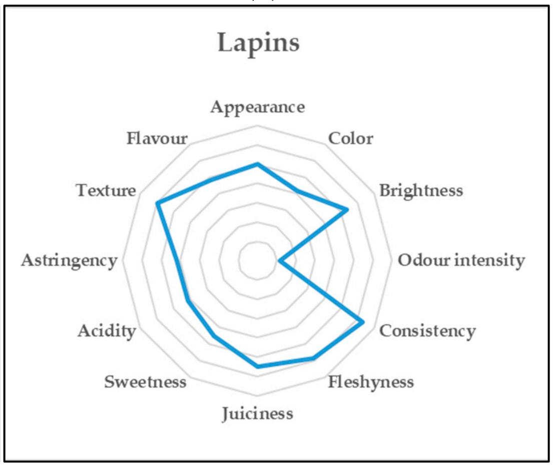

(f)

Table S3. Sensory sheet prepared for the panel test of the four sweet cherry accessions (Mulegnana Riccia, Mulegnana Nera, Montenero and Limoncella) compared to two standard cultivars (Lapins and ‘Ferrovia).

**VISUAL SENSATIONS**		
**Appearance**	○ ○ ○ ○ ○ ○ ○ ○ ○	
**Color**	○ ○ ○ ○ ○ ○ ○ ○ ○<br>**Red__________________Brown**	
**Brightness**	○ ○ ○ ○ ○ ○ ○ ○ ○	
**OLFACTORY SENSATIONS**		
**Odour intensity**	○ ○ ○ ○ ○ ○ ○ ○ ○	
**TASTE/TACTILE SENSATIONS**		
**Consistency**	○ ○ ○ ○ ○ ○ ○ ○ ○	
**Fleshyness of the pulp**	○ ○ ○ ○ ○ ○ ○ ○ ○	
**Juiciness**	○ ○ ○ ○ ○ ○ ○ ○ ○	
**Sweetness**	○ ○ ○ ○ ○ ○ ○ ○ ○	
**Acidity**	○ ○ ○ ○ ○ ○ ○ ○ ○	
**Astringency**	○ ○ ○ ○ ○ ○ ○ ○ ○	
**Peel texture**	○ ○ ○ ○ ○ ○ ○ ○ ○	
**Flavour**	○ ○ ○ ○ ○ ○ ○ ○ ○	
**AFTERTASTE SENSATIONS**		
**Abnormal flavors**	○ ○ ○ ○ ○ ○ ○ ○ ○	
